# Editorial: Novel Endocrine Functions of Bone Marrow Fat

**DOI:** 10.3389/fendo.2019.00349

**Published:** 2019-05-31

**Authors:** Guojing Luo, Li Tian, Janet M. Hock, Xijie Yu

**Affiliations:** ^1^Laboratory of Endocrinology and Metabolism, Department of Endocrinology and Metabolism, and National Clinical Research Center for Geriatrics, West China Hospital, Sichuan University, Chengdu, China; ^2^School of Dental Medicine, Tufts University, Boston, MA, United States

**Keywords:** estrogen deficiency, visceral adipose tissue, endocrinolgy, hematopoietic (stem) cells, bone metastase, bone marrow adipocytes

Bone marrow adipocytes (BMAs) have been neglected when compared to the information available for white, brown, or beige adipocytes. BMAs are the most abundant (and most neglected) cell type in adult bone marrow. Historically, the breakthrough in our ability to study them occurred when technical methods to isolate and culture bone marrow stromal cells were successfully developed by AJ Friedenstein and ME Owen in the 1960–70s. For many years, research focused on the ability of osteoblast precursors to differentiate into either osteoblasts or adipocytes, depending on culture conditions. The BMA remained hidden in plain sight until about 10 years ago, when studies of obesity and anorexia nervosa drew attention to the altered bone marrow environment, and the change in content of BMAs. As we reviewed the literature for this research topic on the role of BMAs, we found considerable variation and inconsistencies. BMAs are puzzling yet intriguing cells, which may yet provide exciting and novel insights into the pathogenesis and treatment of aging, osteoporosis, obesity, aplastic anemia, multiple myeloma, leukemia, and bone metastasis. This stimulated us to establish a research topic within *Frontiers in Endocrinology* to provide a centralized repository of articles and reviews on BMAs.

As a beginning, we present a collection of 3 reviews and 3 research articles. These reviews were focused on the role of BMAs in bone metastasis, hematopoiesis and metabolism. The first review by Luo et al. summarized the potential relationship between BMAs and bone metastasis. *In vivo* and *in vitro* evidences of BMAs strongly suggest an important role in the promotion of bone metastasis. However, most evidence remains indirect and more research is needed. The review by Wang et al. speculated that co-location of BMAs and hematopoietic cells may indicate partnering roles in blood diseases. They reviewed the complex effects of BMAs on blood diseases, especially aplastic anemia, multiple myeloma, and leukemia. The hematopoietic stem cells and its two main branches (myeloid and lymphoid lineage) have close ties with BMAs. The role of BMAs in the pathophysiology in blood diseases continues to be controversial. The endocrine functions of adipocytes have been studied extensively. Such studies have focused mostly on white adipocytes, and not BMAs. The third review by Li et al. summarizes the metabolic feature and endocrine functions of BMAs. One of the oddest characteristics of BMAs is the excessive accumulation of BMAs observed in both energy excess and deficiency conditions. Many mysteries remain in our understanding of the role of BMAs in bone metabolism and energy balance.

The 3 reviews consider historical studies of the negative role of BMAs in perturbing bone homeostasis and normal hematopoiesis, and in enhancing oncogenesis by supporting tumor cell survival. Importantly, we notice an interesting trend over the past 2 years with new studies investigating beneficial effects of BMAs on hematopoiesis and anti-tumor biology. For example, BMAs appear to promote regeneration of haematopoiesis, while PPARγ agonist-induced marrow adipogenesis inhibit leukemia cell growth. The reviews nicely summarize the historical perspective in understanding BMA biology.

The three research articles focused on the potential role of BMAs in the postmenopausal state. Multiple species, including mouse, rabbit, and human, were included in these studies. Ovariectomized and estrogen receptor alpha knockout mice, and ovariectomized rabbits were used as models of the estrogen deficiency of postmenopausal women. The first of these articles by Gavin et al. reported that the production of bone marrow derived adipocytes (BMDA) was enhanced in ovariectomized and estrogen receptor alpha knockout mice, compared to sham controls. Previous studies have indicated that bone marrow derived cells contribute ~10% to the subcutaneous adipocyte population in bone marrow transplantation patients (1). Visceral adipose tissue could not be investigated for ethical reasons. Extending the findings of previous investigations by others, the study by Gavin et al. suggests that the functioning of BMAs may overlap that of visceral adipocytes in an estrogen deficiency state. BMAs accumulation has frequently been associated with bone loss in postmenopausal women.

The article by Li et al. suggests that sclerostin antibody reduces BMAs and promotes bone formation. The Gavin and Li articles showed that abnormal fat accumulation in bone marrow and visceral depot could be correlated with the pathogenesis of postmenopausal osteoporosis and of obesity. The third article by Sollmann et al. reported a positive correlation between ectopic fat deposition in non-adipose tissue (paraspinal muscle) and the bone marrow fat content in the vertebral bodies of postmenopausal woman. This clinical study speculated that abnormal fat distribution in adipose tissue and non-adipose tissue might be linked to the estrogen deficiency state in women. However, correlation is not causation.

It is important to discriminate between cause and consequence in any cell-cell crosstalk. There are challenges in investigating the interdependence of BMA functionality that need to be addressed. The bone marrow microenvironment contains a great variety of cell types and their dynamic interactions will be, extremely complex. Region-specific BMAs have been demonstrated, similar to the heterogeneity of white adipose tissue. The differential contributions of variations in cells and their functional interdependence should to be considered with appropriate experimental design and statistical analyses. Current research has investigated mostly quantity (fat content and distribution) rather than differential functionality and systems biology (paracrine and endocrine functions). While six articles may not provide a full understanding of BMAs, we consider their contribution to the literature as the tip of a huge iceberg! The complexity and systems biology of BMAs calls for more investigation so we can better understand their role in human homeostasis and disease.

To conclude, we sincerely thank all the authors, peer reviews, and editorial board members for their valuable contributions.

## Author Contributions

XY designed this editorial. GL, LT, JH, and XY wrote the manuscript.

### Conflict of Interest Statement

The authors declare that the research was conducted in the absence of any commercial or financial relationships that could be construed as a potential conflict of interest.
